# The Impact of Collaboration Network on Water Resource Governance Performance: Evidence from China’s Yangtze River Delta Region

**DOI:** 10.3390/ijerph18052557

**Published:** 2021-03-04

**Authors:** Hongtao Yi, Yan Yang, Chao Zhou

**Affiliations:** 1 School of Public Administration and Policy, Renmin University of China, Beijing 100872, China; yihongtao@ruc.edu.cn (H.Y.); yang_yan@ruc.edu.cn (Y.Y.); 2 John Glenn College of Public Affairs, The Ohio State University, Columbus, OH 43210, USA; 3 School of Government, Sun Yat-sen University, Guangzhou 510275, China

**Keywords:** collaboration network, governance performance, water governance, China

## Abstract

Existing studies rarely examine the relationship between network structure and network performance. To fill this research gap, this article collects inter-local collaboration network data from 41 cities in the Yangtze River Delta region of China from 2009 to 2015. Based on the institutional collective action framework and social capital theory, we propose bridging and bonding hypotheses regarding the impact of network structures on governance performance. We employ social network analysis and panel data regression models to test the hypotheses. The results show that the coefficients for closeness centrality and clustering coefficient are statistically significant in this analysis, Wuxi played a central role in the collaboration network and the region had formed a close partner network, confirming the positive effect of bridging and bonding network social capital structures on network performance.

## 1. Introduction

In recent years, network research has attracted much attention from public administration and policy scholars, with many existing studies focused on the structure, motivation and performance of the network [1,2,3,4,5,6]. This article selects the Yangtze River Delta local collaboration network as the research object, and explores whether the Yangtze River Delta local collaboration network has a positive impact on water resource governance. Does the internal structure of the local collaboration network in the Yangtze River Delta have a positive impact on water resource governance?

Various network microstructures have been found to drive the formation of collaborative networks [7]. Network structure further influences the interaction between actors in the network, resulting in varying levels of network performance at actor, network and community levels [5,8,9]. Existing studies have comparatively analyzed the dynamic factors shaping network formation in the four estuaries in the United States [10], explored the collaborative mechanisms of the four major city cluster networks in China [6], which include the Beijing-Tianjin-Hebei region [11,12], the Yangtze River Delta region [7], the Pearl River Delta region [13], and the Chengdu Plain urban agglomerations. Scholars have studied the network structure in China and examined the impact of network structure on clean energy development in the United States [5].

Since existing research in the context of China focuses on analyzing the network structure of four major city clusters, three research areas on collaboration network need further exploration. First, the existing network research is mostly using qualitative research methods, with case analysis to explore the dynamics of the network [14,15,16,17], lacking quantitative research on large-N data to examine the impact of network structure on network performance. Although qualitative studies provide evidence about different network structures and explain the mechanisms of influence in collaborate networks, they cannot specify the relationship connecting nodal characteristics and network performance. Second, while scholars pay attention to the empirical contexts of the United States or Western Europe [18,19,20], much less attention has been paid to collaboration network in China. The ICA framework has been well developed in combination with the actual situation in the United States. China has not developed an influential explanatory framework in theorizing the public policy process [21]. Whether the ICA framework can explain the role of collaborative network in China’s environmental governance remains to be tested. Third, scholars focus on the structural characteristics of the network or the driving factors of the network [22,23], explore the effect of collaborative network governance environment and its influencing factors [24]. Scholars pay more attention to how external factors, such as leadership and coordination, affect governance outcomes [24]. However, the internal structure of the network and the performance of the network are rarely linked. Whether the internal structure of the network has an impact in the performance of the network needs further study.

Scholars have proposed that network actors enhance social capital, reduce transaction costs and risks, and promote institutional collective action through bridging and bonding relationships [25,26]. Therefore, we combine the institutional collective action framework (ICA) and the theory of social capital, propose an analytical framework for studying China’s environmental governance practice, analyze the local collaboration network in the Yangtze River Delta of China, and combine with Chinese practices to form an interpretive analytical framework. On the one hand, it tests the applicability of the ICA framework in China, and improves the generalizability of the theory. On the other hand, it contributes to the research perspective of collaborative network and enriches the research on collaborative governance performance.

### 1.1. Literature Review

With economic and social development, increasingly complex public affairs and diversified public service demands have presented challenges for local government governance [7]. Scholars have gradually realized the overall characteristics of inter-regional governance [27,28,29], and proposed new collaborative governance mechanisms such as “collaborative network governance”, “regional collaborative governance”, and “urban agglomerations” [30,31,32,33,34]. “Collaborative governance” is a governance system arrangement. To better formulate and implement public policies and achieve public goals, public institutions and non-state stakeholders are directly incorporated into the formal collective decision-making process [35]. The collaboration between local governments aims to remove the obstacles to regional collaboration caused by administrative borders, such as the formation of the Beijing-Tianjin-Hebei, Yangtze River Delta, and Pearl River Delta local collaboration network, which integrate the resource of multiple regional governments. By formulating consistent goals, the resource allocation of each region is changed from disorder to order, and the coordinated development of the region is promoted [27]. Environmental resource is a type of public resource, which is non-competitive, non-exclusive and has externality [36]. The problem of environmental pollution is closely related to the nature of environmental resource. The decision of a single government will have an impact on other governments, with positive and negative effects. It is difficult for a single government to deal with regional environmental pollution problems alone; therefore, a local collaboration network is formed to jointly carry out the construction of ecological environmental protection [37,38,39].

Scholars pay attention to the structural characteristics of local government collaboration network [1,4,6] and the motivation for the formation of collaboration network [2,3,40]. They point out that institutional and environmental obstacles exist in the collaboration process [41,42], requiring local governments to understand and overcome obstacles to promote effective cooperative governance.

#### 1.1.1. Motivation for Collaboration Network

This study is motivated by the Institutional Collective Action (ICA) framework proposed by Feiock [43,44]. The ICA framework is based on Scharpf’s actor-centered theory and Ostrom’s institutional analysis and development (IAD) framework. It proposes four types of collective action mechanisms: regional organizations, contract network, cooperation groups, and policy network [3]. Regional organizations and cooperation groups are collective actions under multilateral relations. The former relies on legal authority to form legally binding organizational entities, and the latter relies on multilateral agreements to facilitate coordination. The contract network and policy network are management networks featuring bilateral relations. Contract network connects local governments through multi-party agreements, and policy network relies on trust and reciprocity between organizations. Participants in collective actions are local governments and their leaders, who are hypothesized to be rational. Rational agents consider individual and collective interests, and weigh the cost and risk of collaboration [45], when considering whether to participate in cooperative network, and to provide a wider range of public services through collective action. Therefore, ICA pays special attention to the costs and benefits underlying local government officials’ participation in collaboration network [2]. Many scholars have since studied the motivation of collective action among public organizations, focusing on transaction costs [45,46,47] and risks [48,49].

Collective action is constrained by transaction costs, including information coordination costs, negotiation disagreement costs, enforcement supervision costs, and agency costs [2]. Geographical fragmentation, organizational structure, population characteristics, and characteristics of public goods cause heterogeneity in information, preferences and interests among actors in collective action. The higher the heterogeneity, the greater the differences between actors, the higher the transaction costs and the more difficult it is to achieve collaboration [45,50]. Actors adopt a collaborative approach to help reduce transaction costs [46], typically through resorting to formal and informal agreements, information exchange through agreements, and negotiation.

Another motivation affecting collective action is risk. Actors are limited by knowledge, time, resource, and competition. The bounded rationality of policy actors has implications for the collective action outcomes, as they are engaged in a game relationship in the collaborative process. Insufficient information during the collaboration process causes actors to face inconsistent risks. In addition, actors need to setup mechanisms to avoid the risk of betrayal when seeking partners. Berardo and Scholz [48] analyzed the survey data of US estuaries from 1999 to 2001, and found that when solving the relatively simple and low-risk dilemma, actors tended to find popular central actors to expand bridging social capital to reduce transaction risks.

The collective action mechanism through collaboration network will connect multiple actors through formal and informal networks, reducing the risk of betrayal by the actors [51]. The advantages of collaboration network are mainly achieved through institutionalization. Institutionalized network relationships can increase sanctions, enhance mutual trust and mutually beneficial relationships. The network will also provide a monitoring mechanism to identify potential defectors, punish and sanction, and ensure that members keep their commitment to collaboration.

#### 1.1.2. Network Structure and Performance

In recent years, with the development of network research, scholars have gradually focused on the measurement of network performance [52,53]. Regarding the analysis of network performance, there are two mainstream views in academia [54]. One is the research on network environment and network structure [55,56], and the other is the network management and strategy dimensions [57,58,59,60].

Extant literature rarely studies network performance as a dependent variable [61], and most studies focus on explaining network characteristics, or explaining policy effects and public service effectiveness at the organizational level [62]. Provan and Milward [55] offered a preliminary theory to study the effectiveness of the network, and suggested that network effectiveness can be explained by network structure and the network contextual factors. Subsequently, Provan and Sebastian [56] examined the relationship between the network structure of small clusters and network effectiveness, that is, when small cliques are connected, there is a positive correlation between network effectiveness and integration of small clusters. This provided preliminary evidence for the relationship between network structure and network effectiveness, reviving the research on network effectiveness [63]. Turrini [63] further developed the network effectiveness analysis framework, adding network function to the set of influencing factors that determine network effectiveness.

Among the determinants that affect the network effectiveness, scholars hold different views on the impact of network structure. The characteristics of nodes in the network can transform the information flow of the network structure [64]. The network structure focuses on the degree of network integration, including density integration, central integration and cluster integration [65]. Centralized integration of the network helps improve its effectiveness; the integration through a core organization or the proper integration of close links between organizations is less efficient than a fully centralized network [55]. Wang [54] used the fsQCA method to analyze 22 neighboring governance networks in Beijing. The analysis found that centralized network and network effectiveness are negatively correlated, while network density and network effectiveness are positively correlated, indicating that network structural characteristics are neither a sufficient condition nor a necessary condition for the network effectiveness.

The network process management strategy aims to enhance the interaction between network members and promote the cooperation and coordination of network members’ goals and interests [58]. Scholars conducted large-*N* studies, with survey data from the Texas School District [57], and environmental project participants in Taiwan, Spain and the Netherlands [60], to examine the relationship between network management style and network performance. It was found that network management strategies have a positive impact on network performance. The network management strategy enhances trust between members [59], which in turn promotes the exchange of information and knowledge, reducing transaction costs.

Extant literature focuses on the impact of multiple factors such as network structure [66], network function, network context, and network management strategies on network performance. Among them, scholars have different views regarding the influence of network structure [54,55]. The innovation of this study is to use social network analysis methods to analyze network data in the Yangtze River Delta region of China, through examining nodal level network performance for policy actors as embedded in an integrated collaborative network.

#### 1.1.3. Network Governance of Water Treatment

Water resources are a diffused public good, and are non-exclusive. People in the entire river area can use water resources, resulting in negative externalities. For example, residents of the upstream river area cause pollution to the river, affecting water use in downstream area. The pollution costs and consequences resulting from it will be shared by all people who benefit from the area [67]. In addition, the design of administrative region suffers from issues of fragmentation. Local governments consider their own interests in the decision-making under fragmentation. In the decision-making of water treatment, local governments only meet local short-term water demand, ignoring the overall long-term interests of the area, resulting in irrational and inadequate use of water resourcesin the area [68]. Therefore, water resources are generally managed jointly through the establishment of cooperative network [69]. First of all, as a rational entity, local governments consider the costs and benefits of participating in a collaboration network. The network can effectively grasp the information of multiple actors, reduce the cost of dealing with pollution problems, and increase the common benefits of the entire network of actors. Secondly, the problem of water pollution governance is regional. This complex problem is beyond the ability of a single government to solve it. It requires a collaboration network to build a relationship of mutual trust, gather social capital of multiple actors, and jointly solve regional pollution problems [70]. However, collective action easily leads to free-rider problems, making it necessary to strengthen the exchange of social capital between actors to solve the dilemma of collective action. Finally, actors have their own belief systems for water resource management, network of collaboration requires consensus among actors to jointly develop rules and goals for water governance [71]. Studies have focused on important influencing factors in water resource management network, such as institutional characteristics, political culture, the state of water resource ecosystems, geographic proximity, and regional economic development [4,23], which address the collective action dilemma unavoidable in a water pollution management network.

### 1.2. Hypotheses

Network structure promotes cooperation among members through the accumulation of social capital [72]. Social capital was first defined as the sum of social resources. Social members establish relationships through an institutionalized network and share the collective resources owned by the network [73]. From Bourdieu’s definition of social capital, the amount of social capital comes from the whole network and the members themselves, that is, the social resources owned by each member and the total number of social members connected to the network. Coleman [25] developed the definition of social capital and linked social capital with its functions. Social capital is composed of some aspects of social structure and promotes the behavior of social members. Social capital can be conceptualized into characteristics of social organizations, such as reciprocal norms, trust, etc. Mutual trust between members promotes the efficiency of cooperation networks [74].

Cooperative networks will derive different types of social capital. Woolcock and Narayan [75] divide social capital into bridging capital and bonding capital based on the perspective of network structure. Bridging social capital corresponds to a sparse and open network structure. There are structural loopholes between the sparse network and the outside. Burt [72] refers to these structural loopholes as “structural holes”. Network members can establish connections with external resources through structural holes and obtain external capital gains. It is a bridge connecting sparse networks to external relationships. On the other hand, bonding social capital corresponds to a dense and closed network structure. The closed network structure is a structure of strong relationships. Network members are closely connected, which promotes the emergence of effective norms and the creation of trust, thereby enhancing the social capital of members [25].

Bridging social capital and bonding capital play different roles in the network [26]. Network members reduce transaction costs and risks through participating in the collaborate network [23], through which they could enhance their own social capital and improve their status in the network. Regional policy issues often involve multiple different policy individuals, and collective actions are required to resolve regional issues. On the one hand, each policy individual needs to obtain information from the outside to understand and coordinate the interests of all parties in order to achieve its policy goals, which is one of the important motivations for institutional collective action; on the other hand, based on mutual trust between policy individuals, through the establishment of a cooperative network relationship, commitments are made to each other, forming a soft-binding institutionalized collective relationship, and reducing the cooperative risk of collective action [76].

Network structure influences the interaction mode of policy actors in the network and thus the governance effect of the network [5]. In the open network structure, in order to coordinate with multiple actors, the network manager acts as the central actor to coordinate the information exchange and sharing between the actors [56,57]. Since the performance of wastewater treatment is an important goal of the government’s water resource management, local governments attach great importance to wastewater treatment and publish relevant policies and regulations to control wastewater discharge every year. Therefore, in the context of this study, we specifically study wastewater treatment as a governance performance. Wastewater treatment involves information such as factory wastewater discharge and government environmental investment funds. In the local collaboration network in the Yangtze River Delta, each local government owns regional administrative resources and wastewater treatment information. Local government are independent entities and cannot obtain information and resources in neighboring areas. Wastewater treatment issues involve multiple administrative regions. Wastewater discharges into rivers, residents in upstream basins pollute the rivers, which will affect the water use in downstream basins, and the resulting pollution costs and consequences are shared by people of all basins [67]. Each region masters different geographic resources, wastewater treatment information and wastewater treatment goals. The central actor bridges different actors, with better information regarding the wastewater treatment goals of different government in the network. The central actor can transmit, exchange, and share regional governance information and resources, improving the effectiveness of local governments’ grasp of information. It is beneficial to coordinate the target preferences of various regions for wastewater treatment and improve the wastewater treatment performance of the Yangtze River Delta. The central actor has a higher reputation and status, and plays an important bridging role.

The closeness centrality of the network reflects the direct contact between the central actor and other actors, that is, the number of actors that the central actor can directly bridge [1]. The more actors that the central actor can bridge, the better they understand the wastewater discharge status of the Yangtze River Delta and the target preferences of local governments, which is beneficial for coordinating the needs and interests of all parties to jointly deal with wastewater problems.

**Bridging** **Hypothesis:** *The higher the bridging social capital of a network actor, the higher its governance performance*.

In the closed network structure, the actors are in close relationships. Through formal or informal agreements, actors have reached consistency in institutional arrangements and goals, and formed a partnership of mutual trust and mutual benefit [3,77]. Collective actions involve risks such as free-riding and member defections. Since wastewater treatment involves local economic development, strict restrictions on wastewater discharge will affect the rapid development of the local economy. Facing economic and environmental choices, local governments, as rational agents, based on their own interests, might not implement the collective intentions of the cooperative network, and might not want to bear the implementation costs of wastewater treatment, which affects the performance of the entire network. The Yangtze River Delta Local Collaboration Network is a closed cooperation network between local governments. Local governments share information and resources on local wastewater discharge, and form a relationship of mutual trust by signing commitment and implementing actions. In the cooperation network, local governments that kept by their promises have credibility and prestige, which is beneficial for promoting the accumulation of social capital and cooperation between local governments and other individuals [78,79]. The cooperative network has formulated the goals and actions for wastewater treatment. Once members defect to the network and fail to achieve the governance goals, this will affect the reputation and prestige of the defectors. On the one hand, the network will sanction the defectors, and identify defectors who refused to join the partnership.

The network clustering coefficient reflects the number of actors that the network can connect, that is, the network’s ability to solve collective action problems [5]. The higher the proportion of network-connected partners, the higher the prestige and credibility of the average network member, all of which will help increase social capital [80,81]. Once a local government with high social capital violates the collective goal and commits a high discharge of wastewater, it will damage its reputation and bear serious consequences of defecting.

**Bonding** **Hypothesis:** *The higher the bonding social capital of a network actor, the higher its governance performance*.

This article proposes two core hypotheses based on the social capital theory and the ICA framework. Based on testable research hypotheses, we propose a research design. The next section will detail our data collection process and the methods used to analyze the data to further test our core hypotheses.

## 2. Materials and Methods

To test the relationship between the collaboration network and water management outcomes, we collected data from different sources, such as local media and statistical yearbook. City-level data are collected from 41 cities in the Yangtze River Delta region.

### 2.1. Materials

#### 2.1.1. Dependent Variable: Wastewater Treatment Performance

To measure the performance of wastewater treatment in the Yangtze River Delta region, we selected the rate of wastewater treatment from 2009 to 2015 as the dependent variable. The wastewater treatment performance can be measured by the secondary use after the wastewater treatment. Therefore, we collected wastewater treatment rate of the Yangtze River Delta region from statistical yearbook, and chose wastewater treatment rate as the dependent variable.

#### 2.1.2. Independent Variables: Collaboration Network Structure

To test the impact of collaboration network on collaborative wastewater management performance, we collect data to measure network structure related to the hypothesis. First, we searched the local newspaper media and the government official website of all 41 cities in the Yangtze River Delta. With the keyword of “cooperation”, we selected the cooperation agreement between the cities and constructed the network. Second, we coded the network data, with “1” indicating the existence of collaboration between two cities and “0” indicating no collaboration relationship exists between pairs of cities. With the network data, we calculated closeness centrality and clustering coefficient for each city embedded in the network, which are used as key independent variables.

#### 2.1.3. Control Variables

We also included a set of control variables. The rapid population growth, rapid economic development and abuse of water resources have caused the supply of water resources cannot meet people’s needs [82]. The degree of population density is closely related to water pollution [83,84]. Studies have found that population growth causes an increase in organic substances in the water, such as nitrates [83], nitrogen [84] and phosphorus [85]. Therefore, population growth has increased environmental pollution. We collect the number in the population, population density and the rate of population growth data from statistical yearbooks and analyze the correlation between population growth and wastewater treatment.

Scholars have found that there is a correlation between the level of economic development and environmental governance. Economic development will increase the public’s environmental awareness and behavior, which is conducive to the improvement of environmental quality [86,87]. Studies have also found that there is an inverted U-shaped relationship between per capital income and environmental pollution [88], that is, there is an “environmental Kuznets curve” relationship. The increase in the level of economic development has led to an increase in personal and government income. The government can bear the capital investment in environmental governance, and the public will have more time and energy to participate in environmental governance actions, which is useful for improving environmental quality. Therefore, we collect per capital GDP and the rate of GDP growth to measure the level of economic development.

There is also an inverted U-shaped curve relationship between industrial structure and environmental pollution [88,89]. When the industrial structure changes from the primary industry to the secondary industry, environmental pollution will increase. When the status of the secondary industry shifts to the leading role of the tertiary industry, environmental pollution decreases. We measure the industrial structure of the Yangtze River Delta by collecting the proportion of the primary, secondary, tertiary and the number of factories, and analyze the correlation between the industrial structure and wastewater treatment.

In all, we have ten control variables, which are the number in the population, population density, the rate of population growth, GDP, the rate of GDP growth, the proportion of primary, secondary, tertiary, the number of factories and water resource. The data are collected from the China City Yearbook.

Table 1 presents information on variables measure, data sources and the predicted hypothesis results.

### 2.2. Method

Our paper discusses the performance of the collaboration network, and social network analysis method is the main analysis method. In addition, we use UCINET to analyze it. The social network analysis method is used to analyze the behavior and relationship structure between individuals. We explore the correlation between the collaboration network and watershed management performance. Since the data we collected are cross-sectional data from 2009 to 2015, we embedded the network data into the panel data for regression analysis.

#### 2.2.1. Social Network Analysis

The data structure of the social network analysis method is a network structure involving inter-governmental collaboration agreements in 41 regions of the Yangtze River Delta, and belongs to large network data. Therefore, the format presented in UCINET is an edgelist. First, the collaboration relationships concluded between the cities through inter-governmental collaboration agreements are transformed into inter-city collaboration relationships. Then, non-directional measurements are performed on the generated network matrix data, and the closeness centrality of the network and the clustering coefficient are obtained through analysis. Finally, the data are summarized for further analysis.

#### 2.2.2. Empirical Model

To examine collaboration network performance, we need to track data from the same area over a period of time, both cross-sectional data and time dimensions. Therefore, we adopt panel fixed effect regression as the research method. The model can be formulated as follows:

*Y_it_* = *αX_it_* + *βX’_it_* + *z_i_* + *u_t_* (*i* = 1,…,41; *t* = 2009,…,2015)

where *Y* represents the dependent variable, the rate of wastewater treatment from 2009 to 2015 in the Yangtze River Delta region; *X* represents the explanatory variables for the closeness centrality and clustering coefficient for the 41 cities collaboration network from 2009 to 2015; *X*’ represents control variables, including population density, GDP, the proportion of secondary industry and the number of factories. i represents the city, including 41 cities, and *t* represents the year, from 2009 to 2015. *z_i_* represents individual fixed effect and *u_t_* represents time fixed effect.

## 3. Results

We present a descriptive analysis in Table 2 with results based on the data collected. Our observations include 41 cities in the Yangtze River Delta region, and we collect 287 data for 7 the years from 2009 to 2015. Table 2 shows the mean, standard deviation, minimum, and maximum values of the dependent, independent, and control variables. Among them, the minimum value of closeness centrality and clustering coefficient is 0, which indicates that there are two cities with no cooperative relationship among the 41 cities in the Yangtze River Delta region.

Table 3 presents the results of the regression analysis. We used four models to check the assumptions of the regression models. Model 1 tests the effects of economic structure, population density, industrial structure, and the number of factories on wastewater treatment; we included five control variables in model 1. The results show that GDP is significant at the level of 0.01, and shows a positive correlation with wastewater treatment. Meanwhile, population density, the proportion of secondary industry and the number of factories are not significant.

Model 2 only included two core independent variables and a dependent variable; we performed a regression analysis with closeness centrality, clustering coefficient and wastewater treatment. The results show that there is a strong correlation between closeness centrality and clustering coefficient and wastewater treatment. The closeness centrality and clustering coefficient both have a positive correlation with wastewater treatment at a significance level of 0.01.

In model 3, some control variables were added to the model. Model 3 further examines the correlation between the core independent variables and dependent variable. Even though we controlled the influence of population, economic, industrial structure and factory factors, the core independent variables and dependent variable still have a strong and positive correlation. The closeness centrality has a positive correlation with wastewater treatment at a significance level of 0.05, and the clustering coefficient is still at a significance level of 0.01. Among the control variables, GDP is significant at a level of 0.01 and the proportion of secondary industry and the wastewater treatment rate are significant at the 0.05 level. Meanwhile, there is no correlation between population density, the number of factories, and the wastewater treatment rate.

In model 4, we included all control variables. We can see from Table 3 that closeness centrality and clustering coefficient are both significant in model 4. While closeness centrality is significant at the level of 0.1, clustering coefficient is at 0.01. Except for the rate of GDP growth, there is no correlation between the remaining control variables and the wastewater treatment rate. The rate of GDP growth and the wastewater treatment rate is significant at the 0.01 level.

The adjusted R^2^ for four models are 0.18, 0.32, 0.35 and 0.46, indicating that the model fit has improved.

The robustness test results are shown in Table 4. We can see that two major independent variables are still significant. Clustering coefficient remains significant at the 0.01 level. By contrast, the significance level of closeness centrality changed from 0.01 to 0.05, and finally dropped to 0.1 in model 4. Among the control variables, GDP is significant in model 1 and model 3, while there is no correlation between GDP and the wastewater treatment rate in model 4. Additionally, the number of factories is at a significance level of 0.1 in model 1, and the rate of GDP growth is at a significance level of 0.1 in model 4. The remaining control variables aren’t related to the dependent variables. The adjusted R^2^ for the four models are 0.32, 0.35, 0.47 and 0.47, indicating that the models have improved model fit.

**Figure 1:**
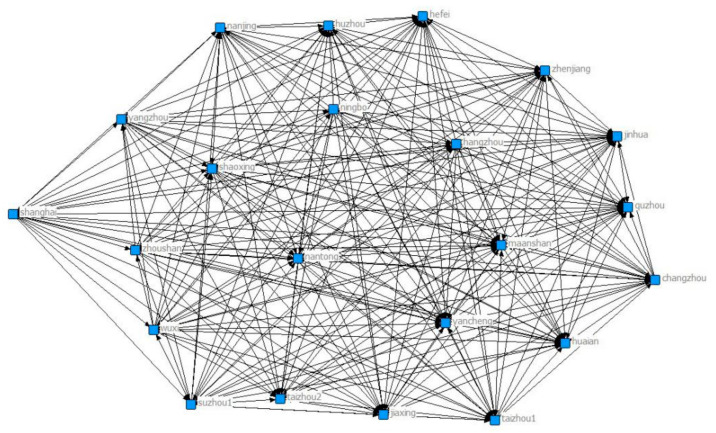
Yangtze River Delta collaborative network in 2009.

**Figure 2:**
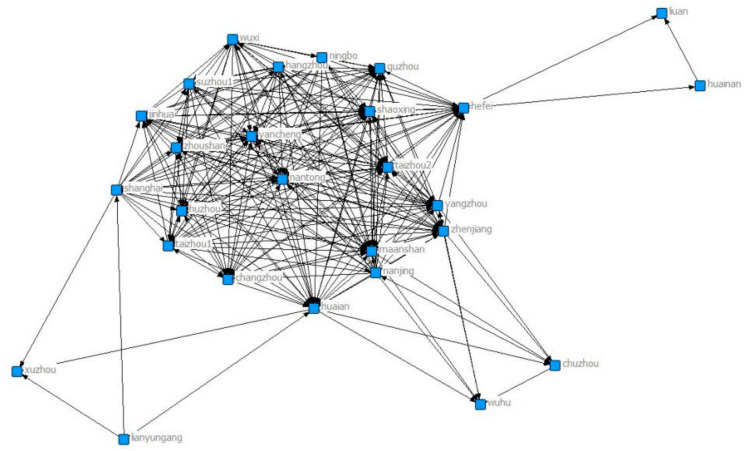
Yangtze River Delta collaborative network in 2010.

**Figure 3:**
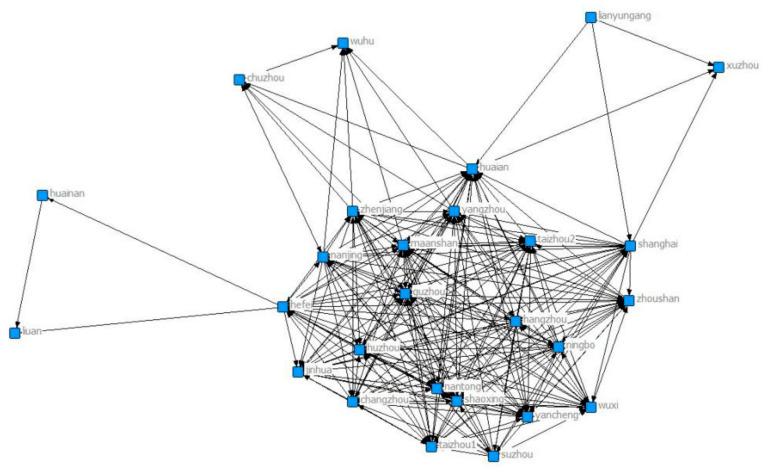
Yangtze River Delta collaborative network in 2011.

**Figure 4:**
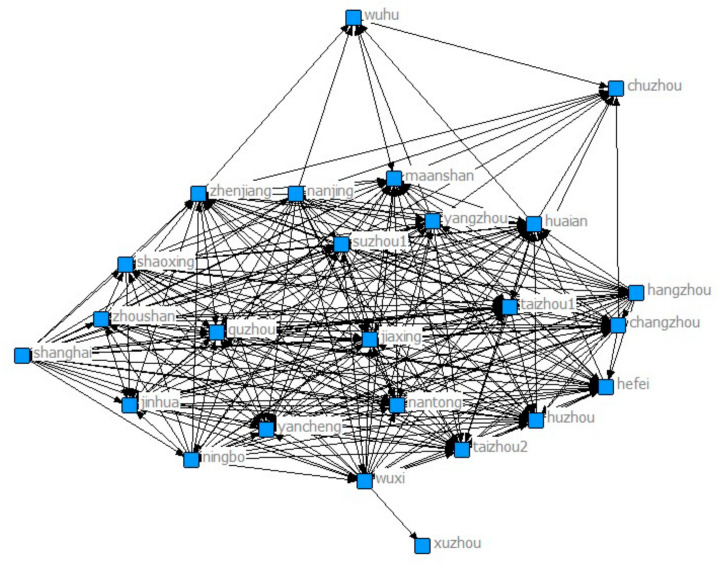
Yangtze River Delta collaborative network in 2012.

**Figure 5:**
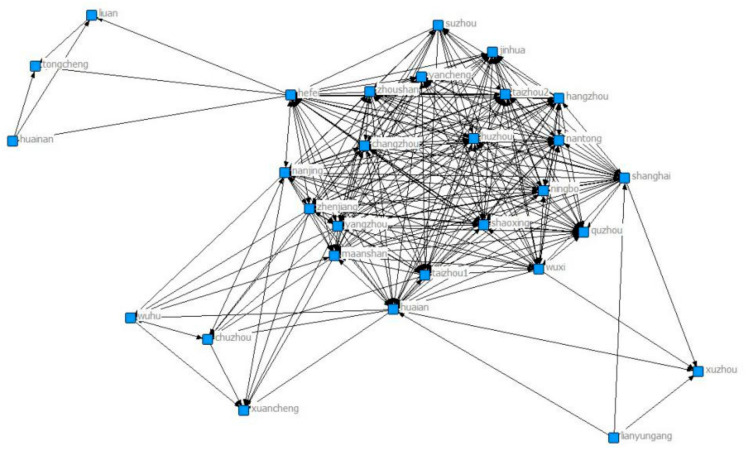
Yangtze River Delta collaborative network in 2013.

**Figure 6:**
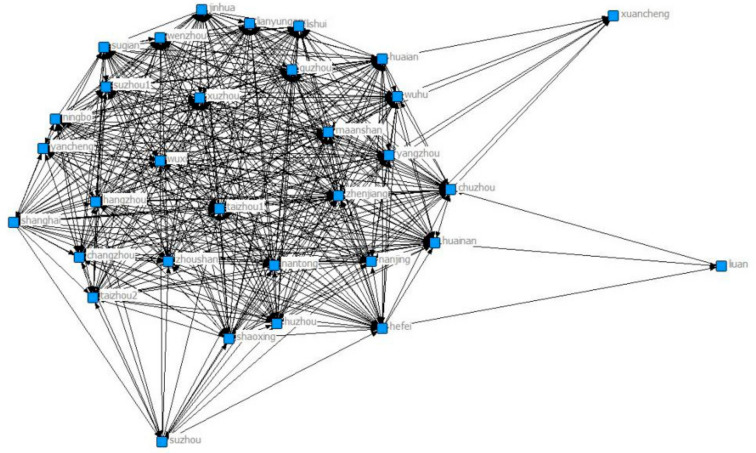
Yangtze River Delta collaborative network in 2014.

**Figure 7:**
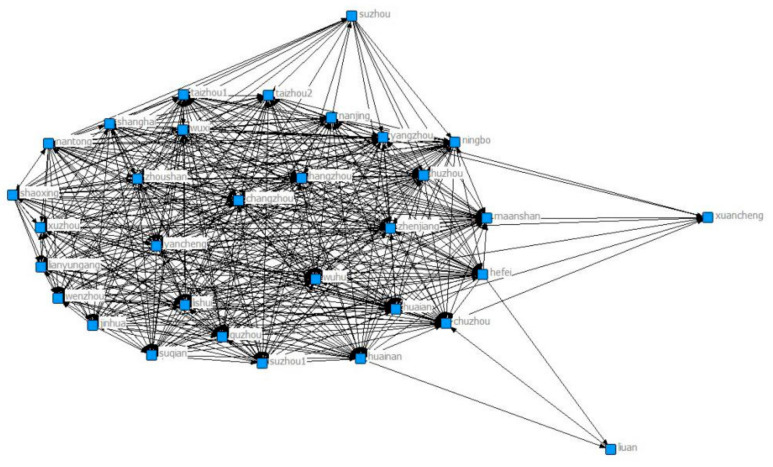
Yangtze River Delta collaborative network in 2015.

Through UCINET visualization, we analyze the closeness centrality and clustering coefficient of collaborate network. Figure 1, Figure 2, Figure 3, Figure 4, Figure 5, Figure 6 and Figure 7 present the corresponding network diagrams. As shown in the figures, there are 41 cities/nodes connected by the collaborative network. In 2009, 22 cities were connected in the network, including Wuxi, Quzhou, Yancheng, Yangzhou, Maanshan, Zhoushan, Zhenjiang, Taizhou1, Shaoxing, Xuzhou, Nantong, Huaian, Changzhou, Lianyungang, Taizhou2, Lishui, Hefei, Hangzhou, Nanjing, Suzhou, Ningbo, and Shanghai. Among them, Wuxi has the largest number of connected cities.

Figure 2 shows that on the basis of the network of 22 cities in 2009, 5 more cities were added in the network in 2010, namely Huainan, Jinhua, Changzhou, Huzhou and Wuhu. Among them, Jinhua, Quzhou, Wuxi, Wuhu have a large number of connected cities.

Figure 3 shows the network diagram for 2011. Through the signing of the collaborative agreements, Chuzhou and Wenzhou joined the network. Wuxi still has the largest number of connected cities, followed by Huainan, Luan, Jinhua and Wenzhou. Wuhu, Chuzhou and Wuxi are more closely linked to other cities.

Figure 4 shows the network in 2012. Compared with the previous years, there was one less city in the collaborative network in 2012, with Yangzhou dropped from participation in the collaborative network in 2012. Wenzhou, Jinhua, Quzhou and Wuxi have the largest numbers of connected cities. Nantong, Huaian, Huainan, Lianyungang, Zhoushan and Wuxi are closely connected with other cities.

Figure 5 shows that two cities were added to the collaborative network in 2013, namely Yangzhou and Xuancheng. There are a total of 30 cities participating in the network. Wuxi, Huainan, Luan and Jinhua have the largest numbers of connected cities, and Wuxi, Xuancheng and Huzhou are more closely connected with other cities in the network.

Figure 6 shows the network in 2014. With respect to the collaborative network of 30 cities in 2013, two new cities were added, namely Suqian and Suzhou. Among them, Luan and Xuancheng become the cities with the largest numbers of connected cities, and Xuancheng and Huzhou are closely connected with other cities in the network.

Figure 7 shows the network diagram in 2015. The number of cities participating in the collaborative network remains unchanged from 32 in 2014. Among them, those with the largest number of cities in the network are more closely linked to other cities.

## 4. Discussion

This article draws its conclusions on the basis of data from the China Yangtze River Delta collaboration network, which shows that there is a central actor in the Yangtze River Delta collaboration network for connecting and coordinating members of the network. If the central actors actively promote interaction between members, the wastewater treatment rate in the Yangtze River Delta region can be significantly improved, and water resource treatment can achieve good results. According to the results of statistical analysis, the central actor in the Yangtze River Delta collaboration network that is most closely connected with other actors is Wuxi. It is located in Jiangsu Province, the hinterland of the Yangtze River Delta plain, and is the transportation center of the Taihu area. Wuxi actively establishes cooperative relationships with other cities in the Yangtze River Delta region, becoming a central actor in the Yangtze River Delta region, mastering a large amount of information and resource, and actively coordinating network members to participate in the process of wastewater treatment. The structural role of network bond social capital is also supported in this paper. The regression coefficient is significantly positively correlated at the level of 0.01, which strongly supports the correlation between the bond structure and the performance of network governance water resource. The analysis results show that a trusting, mutually beneficial and close partner network has been formed in the Yangtze River Delta region, which helps with respect to playing the role of various actors and improving the wastewater treatment situation in the Yangtze River Delta region.

Among the control variables, we found that the level of economic development and industrial structure and the performance of wastewater treatment on the network are related. The level of economic development is mainly measured by the indicators of GDP and the rate of GDP growth. The results show that GDP has a positive effect in the performance of wastewater treatment network in model 1 and model 3, and the rate of GDP growth is negatively correlated with wastewater treatment. The reason may be that the higher the level of economic development, the higher the emphasis on environmental governance. As the most economically developed urban agglomeration in China, the Yangtze River Delta region must not only provide the level of regional economic development, but also pay attention to the governance of the regional living environment. Therefore, collaboration management of regional environmental pollution problems and improvement of regional ecological environment quality have become important goals of the collaboration network. Meanwhile, rapid development of the economy results in severe wastewater discharge. The faster the rate of GDP growth, the worse the governance performance in wastewater treatment. The proportion of secondary industry in the industrial structure also has a significant positive effect on the performance of wastewater treatment in the network. Due to the relatively high proportion of secondary industry, the collaboration network pays more attention to the wastewater treatment of the secondary industry. Members agree on the goals of the agreement, which is conducive to improving the environmental governance of the network. Population density and the number of factories do not affect the performance of wastewater treatment on the network.

## 5. Conclusions

Based on social capital theory and the ICA framework, we propose that bridging network relationships and bonding network relationships have a positive effect on the performance of water resource governance. Through social network analysis and panel data regression methods, we confirmed the hypothesis that the network’s bridging and bonding structures have a positive impact on network governance performance. This research finding makes up for the lack of existing research on network performance, and also provides an empirical basis for the application of the ICA framework in the field of water resource governance in China. The core independent variables of this paper are the closeness centrality and the clustering coefficient of the network. In the regression analysis, the regression coefficient of network closeness centrality is significant at the level of 0.05, which provides strong evidence for the positive effect of network closeness centrality on wastewater treatment.

Existing studies focus on the characteristics of the network, the dynamic factors of network formation, and so on, and rarely focus on the correlation between network structure and network performance. To fill this research gap, this paper collected the inter-governmental collaboration agreements on the environmental field in the Yangtze River Delta region of China, using social network analysis methods and panel regression analysis methods, supports the correlation between network structure and network governance performance. We found that the network relationship of the bridge structure and the bond structure can promote the improvement of the performance of network governance water resource. While Shanghai has the highest level of development, and its economic status is at the core of the region, Wuxi becomes the central actor of Yangtze River Delta collaborate network. The greater the number of actors bridged by Wuxi, the better the network wastewater treatment effect. The greater the number of actors connected by the network, the better the network’s water resource management performance.

Based on the research finding in this article, policy makers can better make regional network development plans and manage the relationships between members in the network. To reflect the role of the collaboration network in managing water resources, policy makers can increase the number of partners connected to the network, expand the resource and information shared by the network, and increase the collective benefits of the collaboration network. At the same time, they actively play the coordination role of central actors, establish close relationships with multiple actors in the form of agreements, mobilize network resources, connect and exchange resource and information of different actors, and strengthen the network’s ability to deal with regional environmental issues.

This paper still has four limitations in terms of data collection and research dimensions, which need to be further developed in subsequent research. First, the representativeness of the data. Due to limited capacity, this article only collects inter-governmental collaboration agreements in the environmental area of the Yangtze River Delta. China still has the Beijing-Tianjin-Hebei region, the Pearl River Delta region, and the Chengdu Plain urban agglomerations. Different urban agglomeration networks have different characteristics and structures. Comparative analysis of performance between networks needs further exploration. Second, based on the social capital theory and the ICA framework, we propose two network structure variables that affect network performance: closeness centrality and clustering coefficient. However, the measurement indicators of network structure are diverse, and the impact of other network structures needs further study. Third, this paper supports the positive role of the collaboration network in wastewater treatment. There are still many problems such as air and soil in the field of environmental governance. Therefore, follow-up research can focus on the governance performance of the collaboration network in other environmental fields. Finally, China is a highly centralized country, under which collaborative networks have their own particular political background. Thus, the performance of network could alternatively be influenced by governance structure, political model and other factors. This could provide a new dimension for further research.

## Figures and Tables

**Table 1:** Variables, measures, and data sources.

Variables	Measures	Predicted Relationship	Data Source
Wastewater management performance (watertre)	The rate of wastewater treatment		China City Yearbook
Bridging social capital (closeness)	Closeness centrality	+	Calculated by author
Bonding social capital (clustering)	Clustering coefficient	+	Calculated by author
Population (pop)	The number in the population	control	China City Yearbook
Population growth (popgro)	The rate of population growth	control	China City Yearbook
Population density (popden)	Population/administrative area	control	China City Yearbook
GDP (GDP)	RMB Ten thousand yuan	control	China City Yearbook
GDP growth (GDPgro)	The rate of GDP growth	control	China City Yearbook
The proportion of the primary (pri)	GDP of the primary/GDP	control	China City Yearbook
The proportion of the secondary (sec)	GDP of the secondary/GDP	control	China City Yearbook
The propotion of the tertiary (ter)	GDP of the tertiary/GDP	control	China City Yearbook
The number of factories (numofidu)	Count	control	China City Yearbook
Water resource (waterres)	City water supply amount	control	China City Yearbook
			

“+” means positive effect.

**Table 2:** Descriptive statistics.

Variables	Obs	Mean	SD	Min	Max
Watertre	287	79.44	12.00	27.27	96.34
Closeness	287	35.40	25.40	0	86
Clustering	287	2.28	2.29	0	10.02
GDP	287	3.06	3.74	2,455,896	251,234,500
GDP gro	287	10.90	2.51	3.74	18.20
Pop	287	501.16	274.26	73.8	1442.97
Popgro	287	4.65	4.57	−2.6	27.4
Popden	287	658.53	342.40	145.29	2275.67
Pri	287	9.61	6.91	0.44	29.7
Sec	287	50.62	7.50	31.81	74.73
Ter	287	39.76	7.49	23.36	67.76
Numofidu	287	3023.54	2945.02	174	17,906
Waterres	287	26,167.84	53,213.03	2172	399,226

**Table 3:** Regression results for the influence of the local collaboration network on watershed management performance.

Variables	Model 1	Model 2	Model 3	Model 4
Closeness		0.11 *** (0.04)	0.09 ** (0.04)	0.07 * (0.04)
Clustering		1.98 *** (0.33)	1.46 *** (0.40)	1.09 *** (0.36)
GDP	5.21 *** (8.00)		2.48 *** (7.96)	7.17 (8.95)
Popden	−0.01 (0.02)		−0.01 (0.02)	−0.01 (0.02)
Sec	0.43 (0.22)		0.51 ** (0.21)	−38.59 (85.71)
Numofidu	0.00 (0.00)		0.00 (0.00)	0.00 (0.00)
GDPgro				−1.17 *** (0.31)
Pop				0.02 (0.03)
Popgro				−0.27 (0.20)
Pri				−40.63 (85.71)
Ter				−38.86 (85.69)
Waterres				−0.00 (0.00)
Constant	45.68 * (18.18)	70.88 *** (1.20)	45.01 *** (16.26)	3972.81 (8569.33)
N	287	287	287	287
Adjusted R^2^	0.18	0.32	0.35	0.46

Standard errors in parentheses, * *p* < 0.1, ** *p* < 0.05, *** *p* < 0.01.

**Table 4:** Robustness test results for the influence of the local collaboration network on watershed management performance.

Variables	Model 1	Model 2	Model 3	Model 4
Closeness		0.11 *** (0.03)	0.09 ** (0.04)	0.07 * (0.04)
Clustering		1.98 *** (0.27)	1.80 *** (0.32)	1.09 *** (0.35)
GDP	5.21 *** (1.45)		2.48 ** (1.06)	7.17 (8.25)
Popden	−0.01 (0.02)		−0.01 (0.01)	−0.01 (0.02)
Sec	0.43 (0.48)		0.51 (0.44)	−38.59 (105.79)
Numofidu	0.00 * (0.00)		0.00 (0.00)	0.00 (0.00)
GDPgro				−1.17 * (0.62)
Pop				0.02 (0.04)
Popgro				−0.27 (0.22)
Pri				−40.63 (106.00)
Ter				−38.86 (106.04)
Waterres				−0.00 (0.00)
Constant	45.68 (29.87)	70.88 *** (1.06)	45.01 * (24.80)	3972.81 (10,597.27)
N	287	287	287	287
Adjusted R^2^	0.32	0.35	0.47	0.47

Robust standard errors in parentheses, * *p* < 0.1, ** *p* < 0.05, *** *p* < 0.01.

## Data Availability

Data sharing not applicable.
